# Neurodevelopmental Outcomes After Neonatal Extracorporeal Membrane Oxygenation (ECMO) in a New ECMO Center

**DOI:** 10.7759/cureus.80020

**Published:** 2025-03-04

**Authors:** Erin Cicalese, Aashish Shah, Jaclynne Nader, Justin Kotliar, Reshma Silas, Sadaf Kazmi, Kristyn Pierce, Purnahamsi Desai, Heather Howell

**Affiliations:** 1 Neonatology, NYU Grossman School of Medicine, New York, USA; 2 Pediatrics, NYU Grossman School of Medicine, New York, USA

**Keywords:** ecmo outcome, neonatal, neonatal intensive care management, neurological outcomes, nicu follow-up

## Abstract

Objective: A standardized multifaceted approach to follow-up is crucial for monitoring neurodevelopment in neonates who undergo extracorporeal membrane oxygenation (ECMO). The Pittsburgh Index for Pre-ECMO Risk (PIPER+) score, which predicts the probability of hospital mortality, may help predict adverse neurodevelopmental outcomes. This study sought to assess the neurodevelopment of neonates who were treated with ECMO in our newly developed ECMO program, by analyzing Bayley Scales of Infant Development (BSID) scores obtained at the Neonatal Comprehensive Care Program (NCCP), our neurodevelopmental follow-up clinic, through two years of age. It also aimed to determine whether neurodevelopmental outcomes in our study population were correlated to PIPER+ score, magnetic resonance imaging (MRI), or video electroencephalography (vEEG) findings.

Study design: We conducted a retrospective chart review of neonatal patients placed on ECMO at our institution between March 2015 and June 2023 who had at least one follow-up visit at the NCCP clinic. The relationships between neurodevelopmental outcomes, quantified by the BSID score, PIPER + score, MRI results, and vEEG abnormalities were analyzed.

Results: A total of 18 patients met the inclusion criteria. There was a significant negative correlation (p<0.05) between PIPER+ and BSID scores at 12 months across all developmental domains analyzed. However, this correlation was no longer significant at 24 months. The odds of the combined outcome of mortality or neurodevelopmental impairment at two years of age increased by 17% for each 1% increase in the PIPER+ score.

Conclusions: Higher PIPER+ scores were associated with higher mortality in our population; they also correlated with worse neurodevelopmental outcomes at 12 months, but not at 24 months. It is important and feasible to follow neonates who underwent ECMO using a neurodevelopmental follow-up clinic.

## Introduction

Extracorporeal membrane oxygenation (ECMO) is a lifesaving, but high-risk cardiopulmonary bypass device that is often reserved for the most critically ill neonates. Infants who have undergone ECMO treatment are at higher risk for neurodevelopmental delays and impairment [1,2]. In 1989, the Extracorporeal Life Support Organization (ELSO) was established to educate, train, and gather data to provide extracorporeal life support throughout the world [3,4]. For the Neonatal Intensive Care Unit (NICU) graduates who underwent ECMO, the ELSO provides general recommendations that serve as a reference guide to support follow-up needs. While some neonatal recommendations trickled down from adult and pediatric literature, more recent studies and retrospective chart reviews have provided a more detailed understanding of neonatal ECMO findings and proposals [5]. Among its recommendations, the ELSO states that NICU ECMO graduates should have neurodevelopmental (e.g., Denver II, Bayley), audiology, and physical/occupational therapy exams and screenings [6,7]. Additionally, if neurological abnormalities are found during any of these evaluations, more frequent follow-up is recommended [8].

In 2016, Maul et al., using data from the ELSO registry, created the Pittsburgh Index for Pre-ECMO Risk (PIPER), which helped determine the effect of certain pre-ECMO variables on survival to hospital discharge. Further modeling on this score, deemed PIPER+, included complications and length of time on ECMO, which helped increase the predictive power of the model [9]. This risk score has not yet been used to help predict neurodevelopmental outcomes. Since ELSO recommendations on follow-up for ECMO patients are nonspecific, insight into the initial PIPER+ score may help determine those infants at high risk for neurodevelopmental impairment. Additionally, while neurological complications can be seen on video electroencephalography (vEEG) and MRI for children who have undergone ECMO, there are no established protocols for screening with these modalities in neonates who have undergone ECMO [10].

The pediatric ECMO program at Hassenfeld Children’s Hospital at New York University (NYU) Langone began in March 2015. The development of this new ECMO program has been described previously [11]. Though an ECMO-specific neurodevelopmental follow-up clinic did not exist when the program began, the NYU Neonatal Comprehensive Care Program (NCCP) clinic established in 1992 was already following the neurodevelopment of infants born prematurely, with critical congenital heart disease, or who had undergone therapeutic hypothermia. We utilized this clinic, with its expertise of neonatologists, occupational therapists, and pediatric psychologists, for follow-up of neonatal ECMO patients.

This study aims to characterize the neurodevelopmental outcomes of neonates who received ECMO at our institution and were seen in follow-up at our NCCP clinic by analyzing Bayley Scales of Infant Development (BSID) scores through two years of age. Furthermore, the study seeks to identify correlations between neurodevelopmental outcomes in this population and PIPER+ score, MRI, or vEEG findings.

## Materials and methods

**Figure 1:**
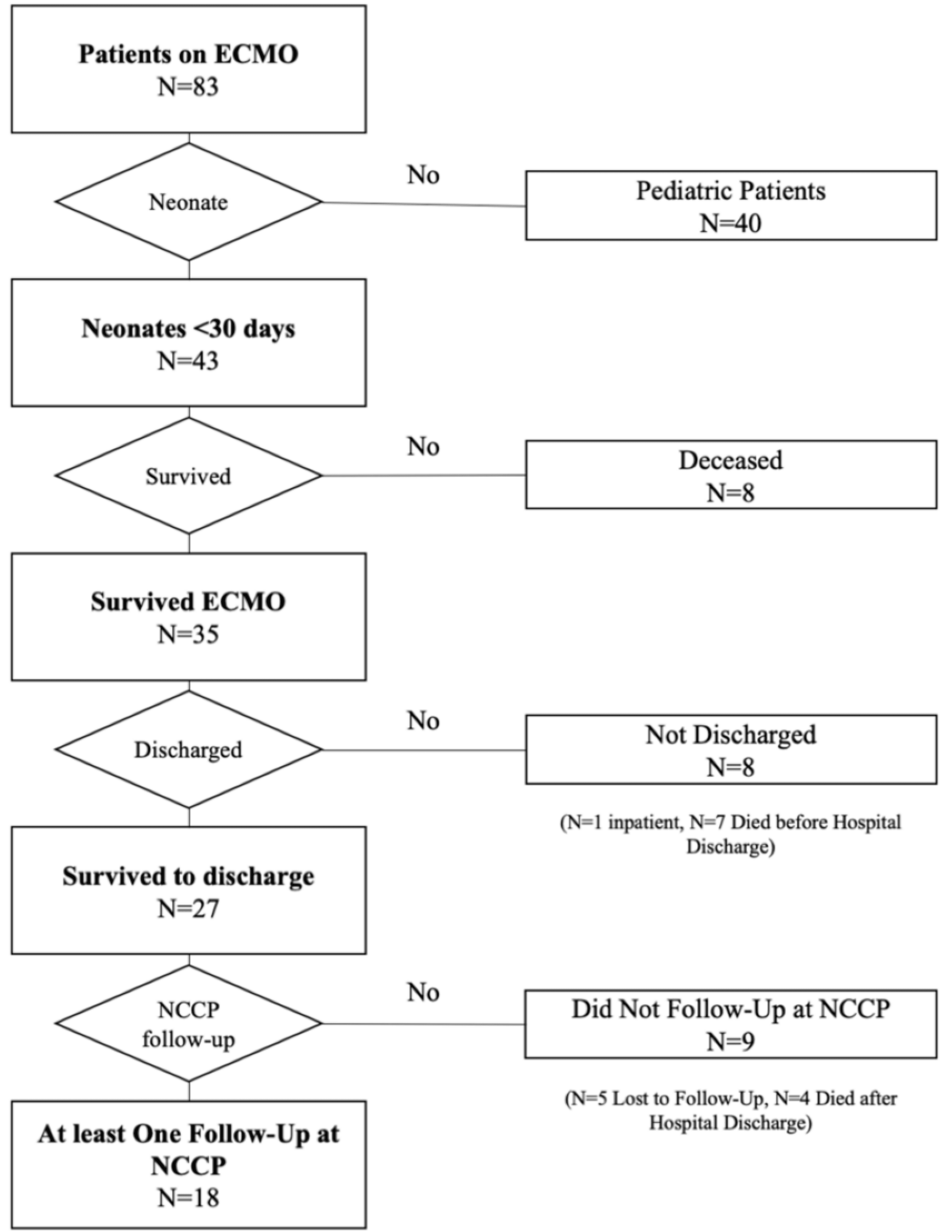
Patient flow sheet

We conducted a retrospective review of all patients who received ECMO between March 2015 and June 2023 and were seen in follow-up at our NCCP clinic. Exclusion criteria included age (neonates defined as <30 days of age), deceased patients, or patients who were not seen at the NCCP clinic. The patient flow sheet can be visualized in Figure 1.

All available data from patients’ 12-month and 24-month follow-up visits were included in the neurodevelopmental analysis. The primary analysis consisted of 18 patients who had 12-month follow-up data available and 12 patients who had 24-month follow-up data available. Analysis of PIPER+ for morbidity included data for all available infants who underwent neonatal ECMO (n=37). Individuals with incomplete data were included in the analysis due to the small sample size and to mitigate bias attributable to complete-case analysis. Statistical analyses were completed using StataCorp 2023 (version 18; StataCorp LLC, College Station, TX).

Patients were classified based on demographics, pre-ECMO mortality risk, ECMO type/duration, the presence of seizures on EEG, and neuroimaging results. Severe radiographic neuroimaging was defined as intraventricular hemorrhages grade III or IV, periventricular leukomalacia, diffuse ischemia, or moderate to large hematomas. Mortality risk was calculated using the validated PIPER+ scale. In addition, hearing impairments were tested by the auditory brain stem response. Neurodevelopmental data were collected using the BSID scores at follow-up. For BSID-III, the normative population mean is 100 for composite scores and 10 for scaled scores. Scores >1 standard deviation (15 points or 1.5 points, respectively) below the mean represent a minor delay, and scores >2 standard deviations (30 points or 3 points, respectively) below the mean represent a moderate to severe delay, which prompts referral to specialists [12].

Demographic and clinical characteristics were summarized for the sample. Anthropometric information and scores for each BSID domain were summarized at each follow-up. Spearman’s correlation coefficient was used to examine the relationship between the PIPER+ score and each domain of BSID-III at the 12- and 24-month follow-up visits. A simple logistic regression was utilized to examine the relationship between the PIPER+ score and the composite outcome of death or neurodevelopmental impairment. Finally, the Mann-Whitney U tests were used to compare scores in each domain of BSID at 12 months to MRI and vEEG results during ECMO admission.

To protect patient privacy, all patient subjects were assigned a study number to de-identify them and anonymously analyze them in a protected and secure database. This study was deemed by the Institutional Review Board (IRB) at NYU to be IRB-exempt.

## Results

Of the 83 pediatric patients who underwent ECMO, 43 were neonates, 35 survived ECMO, 27 survived hospital discharge, and 18 had at least one NCCP clinic visit (Figure 1). Twelve (67%) of the 18 patients who followed up were male. The average gestational age was 39 weeks, and the average birth weight was 3,230 g. Meconium aspiration syndrome was the primary diagnosis in 11 (61%) of the patients, followed by a congenital diaphragmatic hernia in two (11%) of the patients. Eleven (61%) patients were placed on venoarterial (VA) ECMO, six (33%) patients were placed on venovenous (VV) ECMO, and one (6%) of the patients had two separate ECMO runs and was placed on VV and VA ECMO (Table 1).

**Table 1:** Characteristics of the follow-up cohort CHAOS: Congenital High Airway Obstruction Syndrome; ECMO: Neonatal Extracorporeal Membrane Oxygenation

Birth Characteristics	
Gestational age (weeks), mean (SD)	39 (1.7)
Birth weight (g), median (IQR)	3230 (3019, 3685)
Male n (%)	12 (66.7%)
Race	n (%)
White	5 (27.8%)
Black	3 (16.7%)
Hispanic	6 (33.3%)
Asian	2 (11.1%)
Other	1 (5.6%)
Unknown	1 (5.6%)
Neuroimaging/vEEG/ABR	n (%)
Abnormal MRI	5 (of 14) (35.7%)
Abnormal vEEG	6 (of 6) (100%)
Failed ABR	1 (of 14) (7.1%)
ECMO Configuration	n (%)
Venovenous	6 (33.3%)
Venoarterial	11 (61.1%)
Venovenous & venoarterial	1 (5.6%)
ECMO Details	
Age when starting ECMO, days, median (IQR)	1 (1, 2)
Duration, days, median (IQR)	4.5 (3, 6)
PIPER+ score (%), median (IQR)	17 (10, 32)
Primary Diagnosis	n (%)
Meconium Aspiration Syndrome w/ PPHN	11 (61.1%)
Congenital Diaphragmatic Hernia (CDH)	2 (11.1%)
Persistent Pulmonary Hypertension of Newborn (PPHN)	1 (5.6%)
Pulmonary & Tricuspid Atresia	1 (5.6%)
Respiratory Distress Syndrome (RDS)	1 (5.6%)
CHAOS	1 (5.6%)
Tetralogy of Fallot	1 (5.6%)

At 12 months of age, the PIPER+ score had a statistically significant (p<0.05) relationship with BSID scores for cognitive composite, language composite, and motor composite. This relationship was no longer significant at 24 months of age (Table 2 and Table 3). Furthermore, data from all available neonatal ECMO patients showed that the PIPER+ score was statistically significantly (p<0.001) associated with mortality in our patient population, with an odds ratio of 1.075, indicating that, for every 1% increase in the PIPER+ score, odds of mortality increased by 7%. The relationship between PIPER+ and mortality or neurodevelopmental impairment was also statistically significant (p<0.001) with an odds ratio of 1.174. This indicated that, for every 1% increase in the PIPER+ score, the odds of having a combined outcome of mortality or neurodevelopmental impairments at two years of age increased by 17% (Table 4). MRI findings and vEEG findings were not correlated with BSID scores (Table 5).

**Table 2:** Outcomes *1 participant had ASQ instead of BSID-III, 1 patient excluded due to Trisomy 21, 1 patient did not have a formal developmental assessment ASQ: Ages and Stages Questionnaires; BSID: Bayley Scales of Infant Development; SS: standard score

Variable	12-month follow-up (n=18) median (IQR)	24-month follow-up (n=12) medial (IQR)
Age at follow-up (months)	12 (8, 13)	23.5 (18, 25)
Height at follow-up (cm)	73.7 (71.1, 75.6)	84.5 (81.3, 87.6)
Height percentile	47.9 (26.6, 71)	43.3 (6.2, 70)
Weight at follow-up (kg)	9 (8.6, 9.9)	12 (10.7, 13.7)
Weight percentile	53 (36.6, 72.4)	57.9 (9.7, 72)
BSID-III Scores	(n=15) *	(n=8)
Cognitive Composite	100 (95, 110)	102.5 (97.5, 105)
Language Composite	100 (97, 103)	95.5 (94, 101.5)
Receptive Language SS	10 (8, 11)	9.5 (9, 10.5)
Expressive Language SS	10 (9, 11)	9 (9, 10)
Motor Composite	100 (94, 107)	105 (92.5, 112.5)
Fine Motor SS	10 (9, 10)	10.5 (9.5, 11.5)
Gross Motor SS	10 (8, 12)	10 (7.5, 12)
BSID-III Scores < 85	(n=15) *	(n=8)
Language Composite	1 (6.7%)	1 (12.5%)
Motor Composite	3 (20%)	1 (12.5%)
ASQ Result	(n=1)	
Abnormal	1 (100%)	-

**Table 3:** PIPER+ score as a predictor of neurodevelopmental outcomes (N=15) *statistically significant p<0.05 BSID: Bayley Scales of Infant Development

Variable	12-month follow-up (n=15)		24-month follow-up (n=8)	
BSID Scores	Correlation coeff.	p	Correlation coeff.	p
Cognitive Composite	-0.57	0.03*	-0.49	0.21
Language Composite	-0.56	0.03*	-0.09	0.84
Receptive Language	-0.53	0.04*	-0.03	0.97
Expressive Language	-0.35	0.20	-0.30	0.50
Motor Composite	-0.64	0.01*	-0.50	0.21

**Table 4:** PIPER+ as a predictor of mortality or neurodevelopmental impairment *statistically significant p<0.05 PIPER+: Pittsburgh Index for Pre-ECMO Risk

Simple logistic regression of PIPER+ as a predictor of 2-year mortality OR neurodevelopmental impairment (n=37)			
Variable	Odds Ratio (OR)	P value	(95% conf. interval)
PIPER+ score	1.17	0.005*	1.05-1.31
Simple logistic regression of PIPER+ as a predictor of 2-year mortality (n=37)			
Variable	Odds Ratio (OR)	P value	(95% conf. interval)
PIPER+ score	1.08	0.004*	1.02-1.13

**Table 5:** MRI and vEEG as a predictor of neurodevelopmental outcomes *statistically significant p<0.05 BSID: Bayley Scales of Infant Development; IQR: interquartile range; vEEG: video electroencephalography

MRI result as a predictor of neurodevelopmental outcomes at first follow-up (N=11)			
Variable	Normal MRI (n=7)	Abnormal MRI (n=4)	MW test
BSID Scores	Median (IQR)	Median (IQR)	p
Cognitive Composite	105 (95, 110)	95 (87.5, 105)	0.315
Language Composite	103 (97, 112)	101.5 (87.5, 103)	0.461
Receptive Language	10 (10, 13)	9 (7, 10.5)	0.182
Expressive Language	10 (10, 12)	10.5 (7.5, 11.5)	0.818
Motor Composite	103 (94, 110)	101.5 (97.5, 107.5)	0.812
Fine Motor	10 (9, 11)	9.5 (8.5, 11)	0.8
Gross Motor	11 (9, 12)	11.5 (9.5, 12.5)	0.618
Abnormal vEEG as a predictor of neurodevelopmental outcomes at first follow-up (N=15)			
Variable	No vEEG (n=10)	Abnormal vEEG (n=5)	MW test
BSID Scores	Median (IQR)	Median (IQR)	p
Cognitive Composite	100 (95, 110)	100 (95, 100)	0.797
Language Composite	101.5 (97, 103)	100 (86, 103)	0.818
Receptive Language	10 (9, 10)	8 (8, 11)	0.607
Expressive Language	10 (10, 11)	10 (8, 12)	0.979
Motor Composite	100 (94, 107)	95 (77, 103)	0.423
Fine Motor	10 (9, 10)	8 (7, 10)	0.203
Gross Motor	10.5 (9, 11)	8 (5, 12)	0.563

## Discussion

The retrospective review of this follow-up program in a newly formed ECMO center presents evidence of the feasibility of a concerted approach to neurodevelopmental follow-up of complex post-ECMO newborns. In this single-center study, most infants surviving ECMO for the underlying etiologies outlined in Table 1 did well, with BSID scores for cognitive composite at average for both 12- and 24-month follow-up visits. This contrasts with previous studies, which found that patients who undergo ECMO are more likely to have a higher prevalence of developmental delays [12-14]. For example, in a similar-sized pilot study with the leading cause for placement on ECMO being meconium aspiration syndrome, 34% had developmental delay, and 80% of those patients were newborns [15].

PIPER+ scores were created for mortality prediction, and although they are often used clinically to quantify risk to families, they had not previously been used for neurodevelopmental prediction. Our study showed the PIPER+ score continued to correlate with mortality; for every 1% increase in the PIPER+ score, the odds of mortality increased by 7%. We found that neurodevelopmental outcomes also seemed to be related to the complexity of the ECMO patient; for every 1% increase in the PIPER+ score, the odds of having the combined outcome of mortality or neurodevelopmental impairment at two years of age increased by 17%. When broken down further, higher PIPER+ scores correlated with lower BSID scores at 12-month follow-up but no longer correlated at 24-month follow-up. This highlights the importance of closer neurodevelopmental follow-up and provision of therapies as needed after ECMO with catch-up development attainable with early intervention [16]. By 24 months, the association between higher PIPER+ scores and worse neurodevelopmental outcomes disappears. In other words, in our study population, even the most critically ill babies at the time of ECMO cannulation have the potential to achieve normal neurodevelopmental outcomes if they survive ECMO.

The most likely reason for this developmental catch-up is due to the NCCP clinic successfully following through with referrals for early intervention services and subspecialties. A sizable portion of our patients had follow-up services at the time of discharge from the NICU for early intervention services (physical therapy, occupational therapy, and speech therapy), and an even greater amount had subspecialist follow-up. At 12-month follow-up, five more early intervention referrals and three more subspecialty referrals were made. By 24 months, only one new referral was made. This shows the success of the NCCP clinic and the effects of early detection of therapy needs, with patients achieving significant catch-up and either not requiring further therapies or already having appropriate therapies in place.

Many of our ECMO patients underwent an MRI or VEEG, while they were hospitalized. Obtaining an MRI or VEEG is not standard practice for all our ECMO patients; therefore, the data may be skewed towards those with neurological/seizure symptoms or those needing further imaging for prognostication. Despite this, we did not see any correlation between MRI results or VEEG results with BSID scores. This is in contrast to a recent pilot study by Dhar et al., which found abnormal MRI results to be predictive of poor neurodevelopment in a similar patient population [15]. More importantly, this shows that there needs to be standardization regarding practices for MRI and VEEG for prognostication of neurodevelopmental outcomes of ECMO patients. While PIPER+ could be a good proxy metric for long-term outcomes, counseling families with appropriate brain studies similar to what is done for infants who underwent therapeutic hypothermia should become standard practice.

A major limitation of this study is that it is a single-center retrospective chart review. We had a limited sample size and statistical power due to small patient numbers at our new ECMO center, especially in the early years of the program. Factors limiting follow-up included difficulty with transportation between boroughs of New York City, financial constraints, and reluctance to expose their recovering infants to possible communicable diseases for nonemergent reasons, especially during the peak of the COVID-19 epidemic. Furthermore, the transition of appointments to telehealth appointments due to the COVID-19 pandemic interfered with standard follow-up clinic protocol, growth measurements, and administration of BSID. Future directions include refinement to our standardized early follow-up period, including improved communication between the developmental specialists in the hospital, early intervention programs, and subspecialties. There should also be continued education of parents about the importance of follow-up during their hospitalization and potentially a standardized care program to see infants until the age of three or four.

## Conclusions

In conclusion, the establishment of a neurodevelopmental follow-up program for neonates who underwent ECMO at our institution has proven both essential and effective. By leveraging the expertise of the existing NCCP clinic, we have been able to monitor and address potential neurodevelopmental issues in this vulnerable population. This approach aligns with the recommendations from the ELSO, acknowledging the increased risk for neurodevelopmental delays among ECMO survivors. Our findings suggest that, despite the inherent challenges associated with ECMO treatment, many neonates demonstrate favorable neurodevelopmental outcomes when provided with appropriate follow-up and interventions.

Moreover, our study highlights the significance of understanding the relationship between pre-ECMO risk factors, as assessed by the PIPER+ score, and subsequent neurodevelopmental performance. Although we observed that higher PIPER+ scores correlated with decreased BSID scores at the 12-month follow-up, this relationship appeared to diminish by the 24-month mark, suggesting that early intervention and follow-up may foster catch-up growth in neurodevelopment. As we continue to refine our follow-up protocols and expand our patient enrollment, we anticipate further insights that will deepen our understanding of neurodevelopmental trajectories in infants who receive ECMO. Overall, this study underscores the value of structured follow-up programs in improving outcomes for the most critically ill neonates.
